# PALLIATIVE AND END-OF-LIFE CARE TO THE LGBT COMMUNITY

**DOI:** 10.1093/geroni/igz038.2322

**Published:** 2019-11-08

**Authors:** Gary L Stein, Cathy Berkman

**Affiliations:** 1 Wurzweiler School of Social Work-Yeshiva University, New York, New York, United States; 2 Fordham University-Graduate School of Social Services, New York, New York, United States

## Abstract

The lesbian, gay, bisexual, and transgender (LGBT) community experiences discrimination and stigma in accessing health care and social services – including palliative, hospice, and long-term care – across cultures and countries. Health care providers may fail to recognize, acknowledge or address disparities in care. Providers and institutions may be uncomfortable with gender and sexuality issues, and often fail to inquire about sexual orientation and gender identity. It is estimated that there are approximately 2.7 million LGBT adults in the U.S. age 50 and older and approximately 1.1 million age 65 and older. In the UK, an estimated 5-7% of the population identify as LGBT; there are between 600,000 and 840,000 LGBT adults aged 65+. With the projected increased number of older adults and improved treatments that extend the life of seriously ill individuals, even greater numbers of LGBT older adults, and their families, will require palliative and end-of-life in the coming years. Researchers in the US and UK have found that LGBT older adults living in the community and in long-term care facilities experience inadequate, disrespectful, and abusive care due to their sexual orientation and gender identity status. They fear being open about their identities, not receiving equal or safe treatment, and having their family of choice and designated surrogates disrespected or ignored by health care staff. This symposium will describe the experiences of LGBT individuals and their family members, and compare commonalities and differences faced by LGBT communities in the US and UK in accessing palliative and end-of-life care.

